# A possible physiological mechanism of rectocele formation in women

**DOI:** 10.1007/s00261-023-03807-2

**Published:** 2023-02-06

**Authors:** Ge Sun, Robbert J. de Haas, Monika Trzpis, Paul M. A. Broens

**Affiliations:** 1 Anorectal Physiology Laboratory, Department of Surgery, University of Groningen, University Medical Center Groningen, Hanzeplein 1, PO Box 30 001, 9700 RB Groningen, The Netherlands; 2 Department of Radiology, University of Groningen, University Medical Center Groningen, Hanzeplein 1, PO Box 30 001, 9700 RB Groningen, The Netherlands; 3 Division of Pediatric Surgery, Department of Surgery, University of Groningen, University Medical Center Groningen, Hanzeplein 1, PO Box 30 001, 9700 RB Groningen, The Netherlands

**Keywords:** Rectocele, Physiological mechanism, Anal sphincter pressure, Manometry, MRI defecography

## Abstract

**Background:**

We aimed to determine the anorectal physiological factors associated with rectocele formation.

**Methods:**

Female patients (*N* = 32) with severe constipation, fecal incontinence, or suspicion of rectocele, who had undergone magnetic resonance defecography and anorectal function tests between 2015 and 2021, were retrospectively included for analysis. The anorectal function tests were used to measure pressure in the anorectum during defecation. Rectocele characteristics and pelvic floor anatomy were determined with magnetic resonance defecography. Constipation severity was determined with the Agachan score. Information regarding constipation-related symptoms was collected.

**Results:**

Mean rectocele size during defecation was 2.14 ± 0.88 cm. During defecation, the mean anal sphincter pressure just before defecation was 123.70 ± 67.37 mm Hg and was associated with rectocele size (*P* = 0.041). The Agachan constipation score was moderately correlated with anal sphincter pressure just before defecation (*r* = 0.465, *P* = 0.022), but not with rectocele size (*r* = 0.276, *P* = 0.191). During defecation, increased anal sphincter pressure just before defecation correlated moderately and positively with straining maneuvers (*r* = 0.539, *P* = 0.007) and defecation blockage (*r* = 0.532, *P* = 0.007). Rectocele size correlated moderately and positively with the distance between the pubococcygeal line and perineum (*r* = 0.446, *P* = 0.011).

**Conclusion:**

Increased anal sphincter pressure just before defecation is correlated with the rectocele size. Based on these results, it seems important to first treat the increased anal canal pressure before considering surgical rectocele repair to enhance patient outcomes.

**Graphical abstract:**

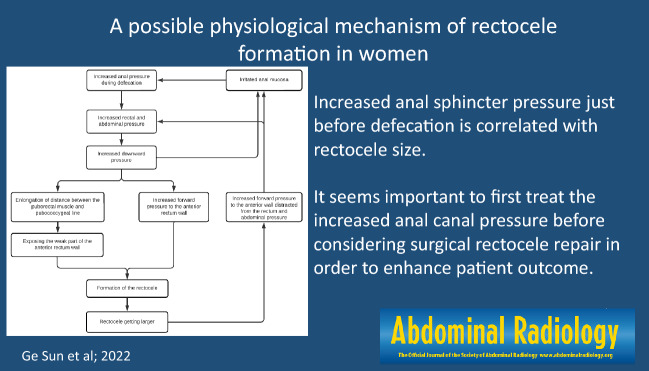

**Supplementary Information:**

The online version contains supplementary material available at 10.1007/s00261-023-03807-2.

## Introduction

By the age of 80, approximately 5% of the female population in the USA will have undergone surgical treatment for rectocele [1, 2]. Unfortunately, the postoperative outcomes are still suboptimal, and high recurrence rates of rectocele and symptoms, especially constipation, have been reported after surgical rectocele repair [3–6]. Thus, the notion that a rectocele and its symptoms can only be solved by surgery seems unjustified [7, 8]. More specifically, surgery merely solves the anatomical problem, while the underlying pathophysiological cause of the rectocele seems to remain untreated. The exact pathophysiology of rectocele formation is still unknown, and this lack of knowledge hampers physicians in deciding on the best treatment strategy for these patients. A 3D computer model showed that increased abdominal pressure might lead to rectocele formation [9]. In another study, a rectovaginal septum defect was related to rectocele formation [10]. In addition, several anorectal physiological factors have been investigated that could possibly be related to rectocele formation, including divergent anorectal pressure [11–17]. Nevertheless, on account of the complexity and multifactorial nature of anorectal physiology, their impact on rectocele formation remains unclear. Some researchers proposed that rectocele formation during defecation was not correlated with anal sphincter pressure during rest [14, 15]. Others reported that paradoxical anal sphincter contraction during defecation might be a risk factor for rectocele formation [11, 12, 18]. Elevated levator muscle pressure during defecation has also been considered as contributing to rectocele development [11, 16, 17]. Lastly, the literature is ambiguous regarding the impact of factors such as age, sex, BMI, and parity on rectocele size [12, 19–25], as well as the influence of rectocele size on constipation [12, 23, 26], and perineal descent severity [15, 27, 28].

To date, no studies have been performed combining manometry measurements, magnetic resonance imaging (MRI), and symptoms to determine the mechanism of rectocele formation. Our aim was therefore to determine which physiological factors may be associated with rectocele formation by combining the findings of anorectal manometry and MRI defecography.

## Patients and methods

### Study population

Initially, we included patients who were referred to the University Medical Center Groningen (UMCG) between 2015 and 2021 because of severe defecation disorders, or because they were suspected of rectocele based on anamnesis and physical examination. We included only these patients who underwent both: the anorectal physiology test at the Anorectal Physiology Laboratory Groningen (APLG) and MRI defecography at the Department of Radiology. These inclusion criteria were met by 60 patients. Exclusion criteria were male sex, severe artifacts at MRI, previous pelvic floor surgery, sacral nerve stimulation potentially influencing anal function, and a time interval between MRI and manometry of more than 12 months. In total, 28 patients were excluded. Information regarding medical history was collected from the electronic patient files. The Groningen Defecation and Fecal Continence Questionnaire (DeFeC), which was validated in a Dutch cohort, was used to evaluate anorectal symptoms, including constipation severity [29]. This questionnaire contains questions about constipation-related symptoms, including straining defecation, hard stools, defecation blockage, and manual defecation. Based on the questions included in the DeFeC, we were able to define constipation according to the Rome IV criteria. The anorectal physiology tests provided information regarding pathophysiological factors underlying defecation disorders.

This study was performed in accordance with the ethical standards of the medical ethical committee of the UMCG (METc 2019/252).

### Magnetic resonance imaging defecography

**Fig. 1:**
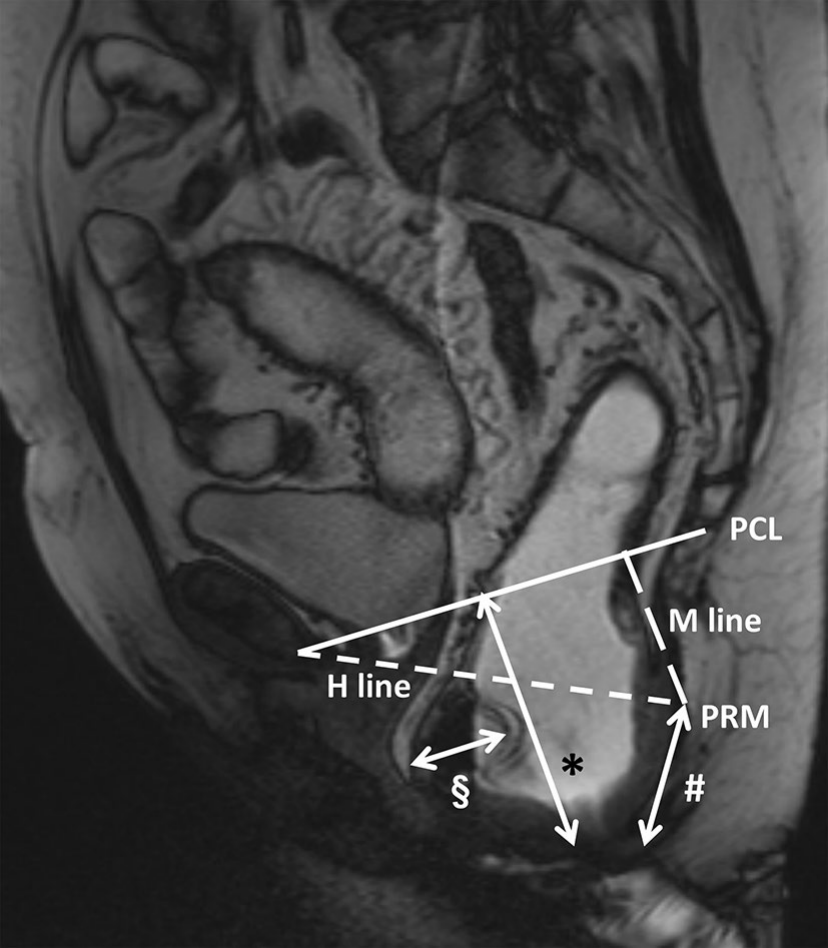
A T2-weighted MRI during the defecation phase in sagittal direction. The parameters used for the analyses in the current study are illustrated in this image. *Distance between pubococcygeal line and perineum; ^#^distance between puborectal muscle and perineum; ^§^rectocele size. *PCL* pubococcygeal line, *PRM* puborectal muscle

Our MRI defecography protocol was based on the protocol proposed in the literature [30], and was used to determine the parameters depicted in Fig. 1. More details regarding our MRI protocol are available in Supplementary Table 1. Rectocele size was determined by measuring the distance between the anterior wall of the rectocele and the normal position of the anterior wall of the anal canal. Perineal descent severity was determined by measuring the distance between the pubococcygeal line and the perineum. The radiological anal canal length was determined by measuring the distance between the perineum and the puborectal muscle. These variables were measured both during rest and during defecation.

### Anorectal physiology tests

The anorectal physiology tests were performed using solar gastrointestinal high-resolution manometry equipment (Laborie/Medical Measurement Systems, Enschede, the Netherlands, Version 9.6), as described previously [31]. No medication potentially affecting the results was taken prior to the tests.

To determine anorectal physiology with manometry, the balloon retention test (BRT) and the defecometry test were used. The BRT was described previously by Jonker et al. [31]. In summary, at our laboratory, the BRT test consisted of introducing two catheters into the anorectum. A catheter (Laborie/Unisensor K14204) with a diameter of 14F with a nonlatex balloon at the tip was inserted into the rectum but not fixed. This rectal balloon was used to mimic solid stool. Later, a Laborie/Unisensor K12981 solid-state (Boston type) circumferential catheter (Laborie Portsmouth NH USA) with an outer diameter of 12F was inserted into the rectum and fixed to the patient’s buttocks, enabling continuous pressure measurement every 8 mm over a total length of 6.8 cm in the anorectum. After positioning, the patient was asked to sit upright on a commode. The balloon was progressively filled with water at body temperature until the patient reported constant sensation, urge sensation, maximal tolerable volume, or until the patient involuntarily lost the balloon before reaching maximal tolerable volume. Following maximal filling of the rectal balloon, it was emptied and the defecometry test started. For this test the rectal balloon was filled with 30–150 mL of water at body temperature. Starting with 30 mL, the patient was asked to defecate the balloon. The volume of water was step-wisely increased with 50 ml until the volume was reached at which the patient would be able to expel the balloon, with the maximal volume at which the patient during the balloon retention test did feel urge sensation. The unfixed catheter with the balloon could be evacuated during the defecometry test.

**Fig. 2:**
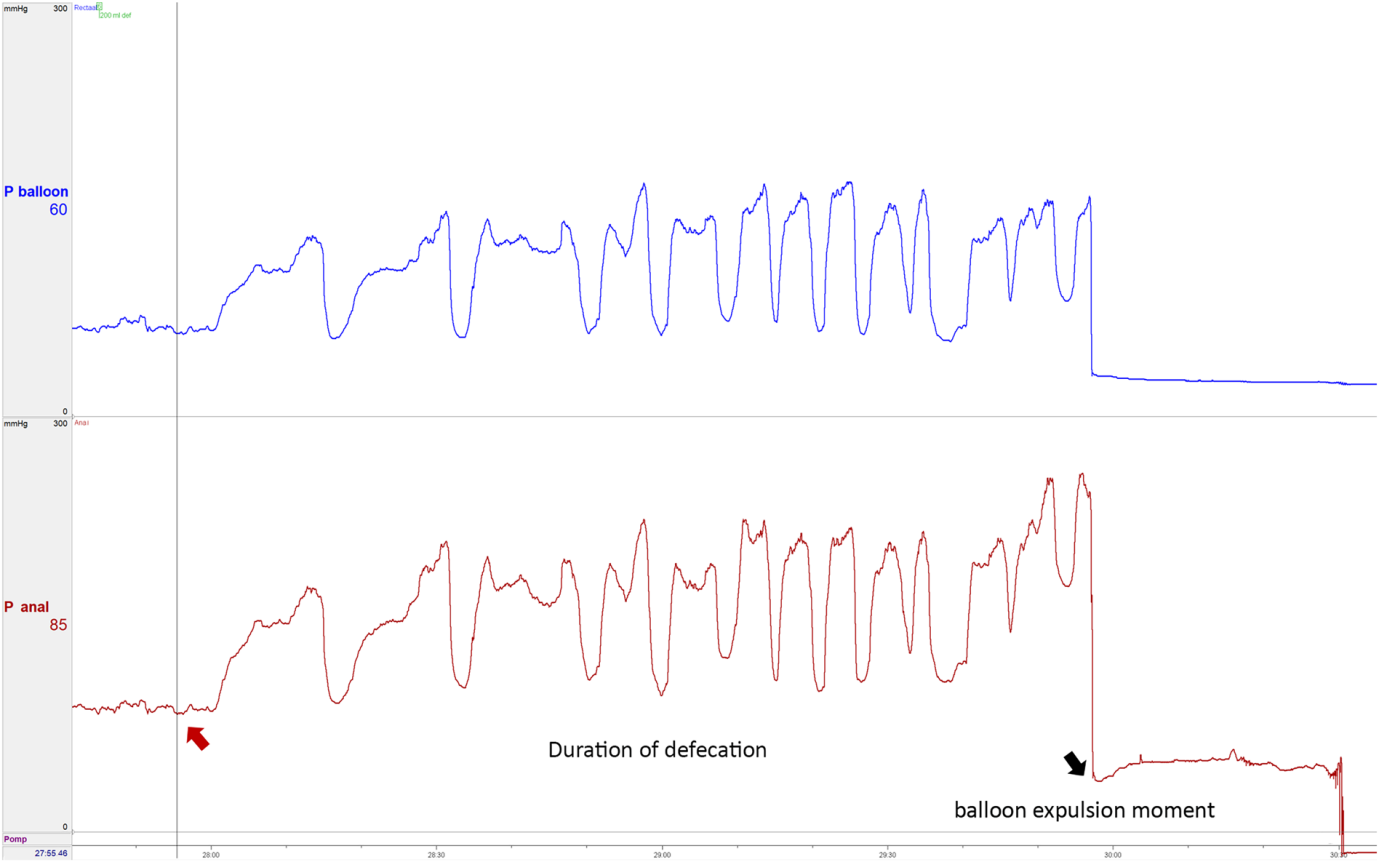
The measurement of the anal sphincter pressure just before defecation. The red arrow indicates the anal sphincter pressure the moment the patient attempts to defecate. The black arrow indicates the moment when the balloon is out. *P balloon* pressure in the rectal balloon, *P-Anal* anal sphincter pressure

The physiological anal canal length was based on the manometry. The volume of the balloon during constant sensation and urge sensation was recorded during the balloon retention test. The compliance of the rectum was calculated based on the results of the balloon retention test at maximal tolerable sensation or maximal retainable sensation. The pressure in the rectum at the level of the puborectal muscle and the anal sphincter was measured during defecation. This included the anal sphincter pressure just before the patient started to defecate the rectal balloon, which is defined as the anal sphincter pressure just before defecation (Fig. 2). Maximal anal sphincter pressure and rectal pressure during defecation were measured, which often occurred just before losing the rectal balloon. To evaluate dyssynergic defecation, we analyzed the function of the anal and puborectal muscles with manometry [32].

The balloon expulsion test was used to evaluate the ability of patients to hold the rectal balloon and defecate. Patients who involuntarily lost the balloon before the end of the test were defined as having severe fecal incontinence, and patients who failed the balloon expulsion were defined as constipated.

### Questionnaire evaluation

The DeFeC questionnaire was completed by 24 of the included patients before manometry. The questionnaire was used to evaluate defecation-related symptoms [29], including constipation and its severity. The severity of constipation was determined with the Agachan constipation score [33].

### Statistical analysis

All statistical analyses were performed using IBM SPSS Statistics, Version 23.0 (Armonk, NY, USA: IBM Corp). Continuous variables were reported as means ± standard deviations and compared with *t* tests when the variables were normally distributed. Associations between two continuous variables were calculated by Spearman rank correlation or Pearson correlation coefficient depending on the normality of data. The normality was tested using Q–Q plots. Linear regression analysis was used to determine potential predictors of rectocele size. A *P* value of < 0.05 was considered statistically significant. Figures were generated using GraphPad Prism 8.2.0 (GraphPad Software Inc, San Diego, CA).

## Results

### Patient characteristics

**Fig. 3:**
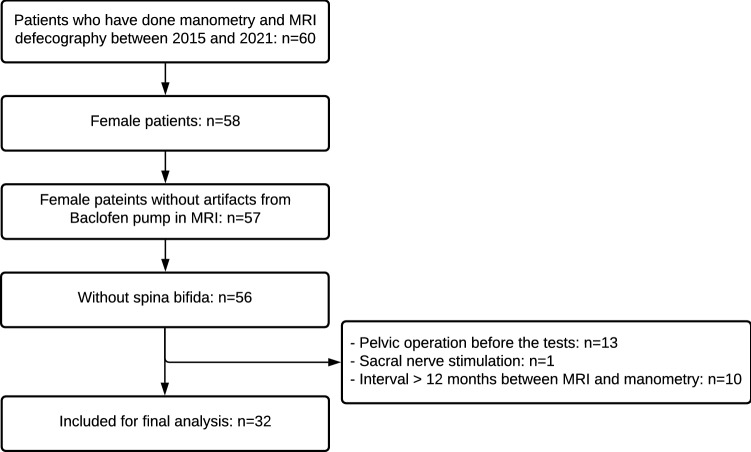
Flowchart of study population

Of the 60 patients selected initially, 32 patients were included in the study for analysis (Fig. 3). The mean age was 48.78 ± 12.70 years. The mean body mass index was 25.81 ± 5.14 kg/m^2^. The median time between MRI and manometry was 4.5 months (range 0–12). Additional patient characteristics are presented in Supplementary Table 2.

### Magnetic resonance imaging defecography

At MRI, 30 (94%) patients had a rectocele. During rest, the mean rectocele size at MRI was 1.07 ± 0.44 cm, while during defecation this was 2.14 ± 0.88 cm. The distance between the puborectal muscle and the perineum during defecation was 3.07 ± 0.87 cm, and the distance between the pubococcygeal line and the perineum during defecation was 6.85 ± 1.39 cm.

### Manometry

Just before starting defecation, the mean rectal pressure, mean puborectal pressure, and mean anal sphincter pressure were 37.96 ± 19.67 mm Hg, 37.67 ± 23.97 mm Hg, and 123.70 ± 67.37 mm Hg, respectively (Table 1). Using the expulsion test we found that out of the 32 patients, one lost the balloon before the test was started, two were not able to expel the balloon, and the other 29 patients were able to expel the balloon. Out of all the 32 patients, 30 had confirmed dyssynergic defecation, and only one patient did not experience this symptom. For one patient, the data needed for the diagnosis of dyssynergic defecation were missing.

**Table 1:** Summary of measurement of manometry and MRI

Anorectal manometry test variables	
Physiological anal canal length (cm)	3.54 ± 0.63
Rectal pressure just before defecation (mm Hg)	37.96 ± 19.67
Puborectal pressure just before defecation (mm Hg)	37.67 ± 23.97
Anal sphincter pressure just before defecation (mm Hg)	123.70 ± 67.37
Rectal maximal pressure during defecation (mm Hg)	109.82 ± 78.95
Puborectal maximal pressure during defecation (mm Hg)	108.78 ± 45.66
Anal sphincter maximal pressure during defecation (mm Hg)	190.44 ± 96.10
Dyssynergic defecation	
Yes	30
No	1
Missing data	1
Rectal compliance (ml H_2_O/mm Hg)	7.45 (5.13 – 20.38)
Constant sensation volume (mL)	100 (41 – 175)
Urge sensation volume (mL)	155 (93.75 – 327.5)
MRI variables	
Distance between puborectal muscle and perineum during defecation (cm)	3.07 ± 0.87
Distance between pubococcygeal line and perineum during defecation (cm)	6.85 ± 1.39
Rectocele size during defecation (cm)	2.14 ± 0.88

Continuous data were expressed as mean ± SD or median and interquartile ranges

### Relation between rectocele characteristics and anorectal physiology

**Fig. 4:**
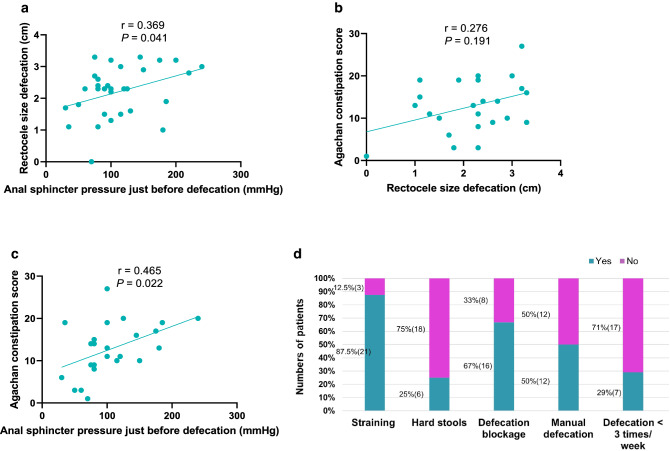
The relationship between rectocele size and anal sphincter pressure at the start of defecation (**a**), between the Agachan constipation score and rectocele size (**b**), between the Agachan constipation score and anal sphincter pressure at the start of defecation (**c**), and the prevalence of constipation-related symptoms (**d**)

Anal sphincter pressure just before starting defecation was moderately and positively correlated with the rectocele size (*r* = 0.369, *P* = 0.041, Fig. 4a), and it strongly predicted rectocele size when using linear regression analysis (*β* = 0.006, 95% CI < 0.001–0.011, Table 2). In contrast, during rest, anal sphincter pressure, puborectal muscle pressure, and rectal pressure were not correlated with rectocele size during rest (*P* > 0.05 in all instances).

**Table 2:** Univariable linear regression analyses of factors which might influence rectocele size measured during defecation

Independent variables	*B*	Beta coefficient	95% CI of *B*		*P*
			Lower bound	Upper bound	
Demographic factors					
Age (years)	− 0.007	− 0.098	− 0.033	0.019	0.594
BMI (kg/m^2^)	0.002	0.014	− 0.066	0.071	0.941
Vaginal delivery	0.421	0.217	− 0.299	1.142	0.241
Cesarean section	0.277	0.117	− 0.653	1.207	0.547
Traumatic births	0.494	0.217	− 0.197	1.185	0.154
Anorectal physiological factors					
Physiological anal canal length (cm)	0.060	0.041	− 0.486	0.607	0.823
Rectal pressure just before def (mm Hg)	0.004	0.067	− 0.02	0.028	0.727
Puborectal pressure just before def (mm Hg)	0.001	− 0.005	− 0.011	0.011	0.978
Anal sphincter pressure just before def (mm Hg)	0.006	0.369	< 0.001	0.011	***0.041***
Maximal rectal pressure def (mm Hg)	0.001	0.136	− 0.002	0.005	0.474
Maximal puborectal pressure def (mm Hg)	0.003	0.216	− 0.003	0.009	0.288
Maximal anal sphincter pressure def (mm Hg)	0.002	0.256	− 0.001	0.004	0.165
Rectal compliance (ml H_2_O/mm Hg)	− 0.003	− 0.058	− 0.026	0.019	0.763
Constant sensation volume (mL)	< 0.001	0.035	− 0.003	0.003	0.858
Urge sensation volume (mL)	0.001	0.210	− 0.001	0.003	0.303
MRI factor					
Radiological anal canal length def (cm)	0.011	0.103	− 0.027	0.048	0.574

Significant differences (*P* < 0.05) are indicated in bold

Beta coefficient: standardized coefficients

Def: defecation

During defecation, there were 15 patients having rectocele below the levator, 10 patients having rectocele at the level of the levator, 5 patients above the levator, and 2 patients having no rectocele. There is no significant difference in anorectal physiology between the patients with rectocele located below the levator during defecation and rectocele located at or above the level of levator ani muscle (all *P* > 0.05).

### Relation between rectocele size and constipation

The mean Agachan constipation score was 12.79 ± 6.28. The Agachan constipation score was not significantly correlated with rectocele size during defecation (*r* = 0.276, *P* = 0.191, Fig. 4b), but moderately correlated with anal sphincter pressure just before defecation (*r* = 0.465, *P* = 0.022, Fig. 4c).

Patients in the current study mostly reported straining (87.5%) and defecation blockage (66.7%) (Fig. 4d). Anal sphincter pressure just before defecation started was correlated with straining (*r* = 0.539, *P* = 0.007) and blockage (*r* = 0.532, *P* = 0.007), but did not correlate with hard stools, the need for manual defecation, or stool frequency (Table 3). None of the above-mentioned symptoms was associated with rectocele size during defecation.

**Table 3:** The correlation of constipation-related symptoms and the rectocele size and anal sphincter pressure just before defecation

		Straining*	Hard stools*	Defecation blockage*	Manual-supported defecation*	Stool frequency < 3 times a week*
Anal sphincter pressure just before defecation (mm Hg)	*r*^#^	*0.539*	− 0.1	*0.532*	0.212	− 0.16
	*P*	***0.007***	0.721	***0.007***	0.321	0.456
	*N*	24	24	24	24	24
Rectocele size during defecation (cm)	*r*^#^	0.174	0.035	0.013	− 0.181	0.134
	*P*	0.417	0.871	0.953	0.396	0.529
	*N*	24	24	24	24	24

Significant differences (*P* < 0.05) are indicated in bold

^#^Spearman rank correlation test

^*^Collected using the Groningen Defecation and Fecal Continence questionnaire

Additionally, neither age, BMI, vaginal delivery, cesarean section, nor traumatic births predicted rectocele size during defecation (Table 2).

In 10 out of 32 patients (31%), information about the precise duration of symptoms was available. The Spearman test did not show a significant correlation between duration of symptoms and rectocele size (*r* = 0.563, and *P* = 0.09).

### Relation between rectocele formation and perineal descent severity

**Fig. 5:**
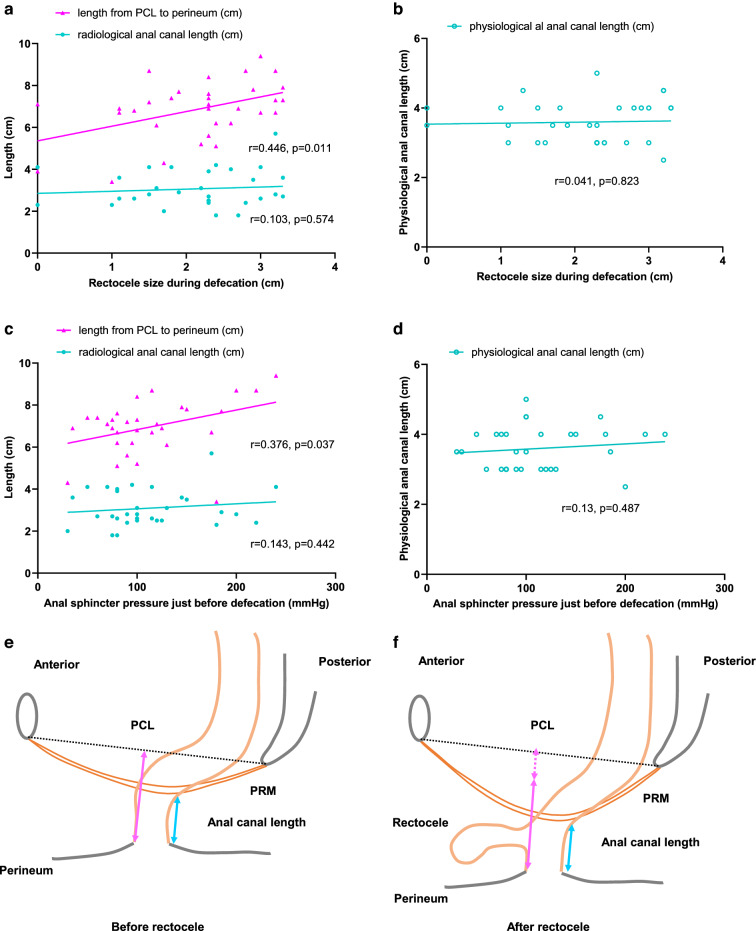
The relation between rectocele size during defecation and the distance between the pubococcygeal line and the perineum at MRI and radiological anal canal length (**a**), and physiological anal canal length during manometry (**b**). The relation between anal sphincter pressure just before defecation and the distance between the pubococcygeal line and perineum at MRI and radiological anal canal length (**c**), and physiological anal canal length measured with manometry (**d**). Illustration of the descent of the anal canal (or perineal descent, the red line indicates the distance between the pubococcygeal line and perineum, and the blue line indicates the anal canal length) from normal condition without rectocele (**e**) to after rectocele formation (**f**)

The distance between the pubococcygeal line and perineum was positively correlated with rectocele size (*r* = 0.446, *P* = 0.011, Fig. 5a). Radiological anal canal length and the physiological anal canal length were not correlated with rectocele size during defecation (Fig. 5a, b).

The anal sphincter pressure was positively correlated with a larger distance between the pubococcygeal line and perineum (*r* = 0.376, *P* = 0.037, Fig. 5c), but was not correlated with either the radiological anal canal length (Fig. 5c) or the physiological anal canal length measured with manometry (Fig. 5d).

## Discussion

In this study, we found that increased anal sphincter pressure just before starting defecation correlated with a larger rectocele during defecation and with severe constipation.

**Fig. 6:**
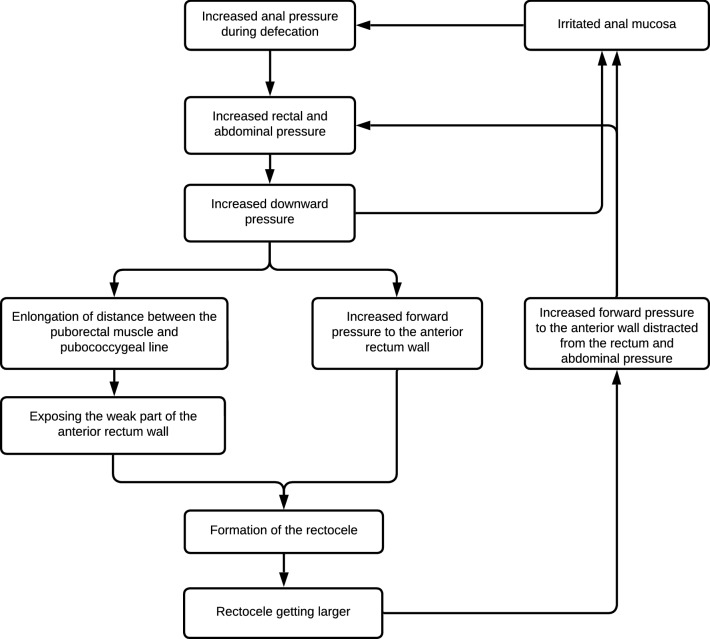
Schematic illustration of our proposed mechanism of rectocele formation. Increased anal sphincter pressure during defecation requires increased rectal and abdominal pressure by pushing. This leads to higher downward pressure. Straining pushes the anal canal caudally, thus exposing the weak part of the anterior rectal wall. This contributes to the formation of the rectocele. As the rectocele increases in size, more pressure is distracted to the anterior direction and the downward-directed strength decreases. This requires further increased rectal and abdominal pressure by harder pushing. Feces blocked by high anal canal pressure will become drier and defecation of that will damage the anal mucosa and lead to hypersensitivity of the contact receptors of the anal external sphincter continence reflex, resulting in over-action of the reflex and spasm of the external anal sphincter. This results in increased anal basal pressure [36]. So the patient is caught up in a vicious circle

Based on our results, we propose the following hypothesis regarding rectocele formation (Fig. 6). We believe that the patient needs to strain hard because of increased anal sphincter pressure during defecation, i.e., the anal sphincter pressure just before starting defecation. This may lead to increased abdominal pressure [34], which would corroborate the findings of others that there is an association between increased abdominal pressure and rectocele formation [9]. The increased pressures will then push the anal canal caudally, stretching and weakening the anterior rectal wall as a consequence. Meanwhile, the forward-directed pressure is increased, and the anterior rectal wall protrudes forward, leading to an anterior rectocele. We hypothesize that when the rectocele increases in size, more pressure will be distracted forward in female patients, leading to a decrease in downward pressure. Patients will strain harder to increase the downward pressure. During straining, feces may irritate the anal mucosa leading to hypersensitivity of the contact receptors of the anal external sphincter continence reflex [35], which in turn results in increased anal sphincter pressure [36], and the patients find themselves caught up in a vicious circle. Although the current study was only based on female patients, we believe that rectovaginal septum weakness does not play a major role in rectocele formation as illustrated by our proposed mechanism in Fig. 6. Therefore, our hypothesis on rectocele formation could also be applicable to male patients.

In this study, rectocele size was correlated with the anal sphincter pressure just before starting defecation, which is concordant to the findings of Johansson et al. [11]. They suggested that paradoxical sphincter contraction increases anal sphincter pressure during defecation [37], and that this may be a causative factor in rectocele formation [11]. Ambiguous outcomes regarding the contribution of anal pressure to rectocele development probably result from the fact that different studies measured pressures in different physiological conditions [11, 14, 15]. According to Buyukasik et al. [14] and Yoshioka et al. [15], anal sphincter resting pressure is not correlated to rectocele formation. This is probably because they measured the pressure in the anal canal at rest with the rectum empty instead of filling the rectum until the patients were able to expel the balloon, as we did in our study. According to the literature, anal resting pressure mainly contributes to fecal continence during rest [38], while anal sphincter pressure is increased during defecation because of damaged anal mucosa as a result of pushing stool through a narrow anal canal by dyssynergic defecation [39].

Interestingly, in our group of patients, the anal sphincter pressure just before defecation was not only correlated with rectocele size but also with the constipation score and constipation symptoms (straining and blockage during defecation), while the rectocele size itself was not correlated with these symptoms. This finding is consistent with Wexner et al., who reported that constipation in rectocele patients is associated with the disability of the pelvic floor muscles to relax [40]. Thus, constipation in patients with a rectocele is probably not caused by the anatomical abnormality, which was assumed to be responsible for defecation disorder [25]. Instead, it is the increased anal sphincter pressure just before defecation which hampers proper relaxation or leads to the paradoxical contraction of the anal sphincter, and consequently, it impairs the defecation process. Additionally, dry feces blocked by increased anal canal pressure just before defecation will damage the anal mucosa and lead to hypersensitivity of the contact receptors of the anal external sphincter continence reflex [35], resulting in overreaction of this reflex and spasm of the external anal sphincter [36]. This will result in increased anal sphincter pressure just before defecation [36]. Surgical rectocele repair alone will not end this vicious circle of increased anal pressure. Therefore, given the high postoperative recurrence rates [3, 4], surgical rectocele repair should not be the only treatment option considered to relieve patients of their constipation symptoms. A potential way to end the vicious circle of rectocele formation could be a botulinum toxin injection to decrease anal sphincter pressure [41], and biofeedback therapy or pelvic floor physical therapy can be utilized to counter dyssynergic defecation [8], and to avoid rectocele development or worsening.

We found rectocele size during defecation to be correlated with the distance between the pubococcygeal line and perineum instead of radiological or physiological anal canal length. This indicates that larger rectocele size is correlated with descent of the anal canal (Fig. 5e, f) and is consistent with the previous reports that rectocele and perineal descent often accompany each other [15, 27], and might be explained by the observation that rectocele size and perineal descent are both correlated with increased anal sphincter pressure just before defecation. The straining maneuver pushes the anal canal in caudal direction and increases the distance between the anorectal junction and the pubococcygeal line.

Information about the exact duration of symptoms was missing in a considerable amount of patients because these patients were referred to our hospital due to resistant and recurrent symptoms of constipation. Due to the relatively small sample size, we cannot draw firm conclusions regarding the correlation between symptom duration and rectocele size.

There are some limitations to this study that need to be mentioned. First, this was a retrospective study and therefore, some data were missing. The retrospective character of the study also partially contributed to the fact that we were able to investigate outcomes of patients with only relatively small rectocele, which is the second limitation of this study. This limitation was also potentially caused by our strict inclusion criteria, as we included only these patients who had undergone both MRI and manometry. In our hospital, however, it is standard care that patients with simple and obvious rectoceles are not referred to both MRI and manometry. This limitation might bias the generalizability of our results. However, we believe our findings will be of importance for clinicians involved in treating patients with defecation disorders not fully understood by anamnesis and physical examination. A prospective study is needed to further evaluate our finding in larger patient series with a wider range of rectocele sizes. Third, several patients in the current study had a rectocele smaller than 2 cm, which could have influenced the correlation between the anal sphincter pressure just before defecation and the rectocele size. However, the correlation remained significant, which seems to indicate that the pathological mechanism underlying rectocele development should already be corrected in early stage relatively small rectoceles. Fourth, our study population was relatively small, partially due to our exclusion criteria that led to having to exclude a significant number of patients. Nevertheless, we believe that our exclusion criteria were necessary to obtain a homogeneous study population that enabled us to draw well-founded conclusions.

## Conclusion

Anal sphincter pressure just before starting defecation is correlated with larger rectocele size and constipation-related symptoms. However, no clear relation exists between severity of constipation and rectocele size. Based on these results, it seems important to first treat the increased anal canal pressure before considering surgical rectocele repair to enhance patient outcome.

## Supplementary Information

Below is the link to the electronic supplementary material.

Supplementary file1 (DOCX 17 kb)

Supplementary file2 (DOCX 16 kb)

## Abbreviations

MRI: Magnetic resonance imaging
DeFeC: Groningen Defecation and Fecal Continence Questionnaire
BMI: Body mass index
BRT: Balloon retention test
PCL: Pubococcygeal line
PRM: Puborectal muscle
