# Supplementary material for: A possible physiological mechanism of rectocele formation in women
Source: Abdom Radiol (NY). 2023 Feb 6;48(4):1203–14. doi: 10.1007/s00261-023-03807-2 (PMC10115871; doi:10.1007/s00261-023-03807-2)
Supplement: Supplementary file 1 — Supplementary file1 (DOCX 17 kb) [file 261_2023_3807_MOESM1_ESM.docx]

|  | **Static 2D MR sequences** | | | **Dynamic 2D MR sequences** |
| --- | --- | --- | --- | --- |
|  | **T2 TSE axial** | **T2 TSE sagittal** | **T2 TSE coronal** | **T2 TruFi sagittal*** |
| Matrix | 260x384 | 227x384 | 189x320 | 189x320 |
| Slices | 45 | 27 | 33 | 1 |
| Slice thickness | 4 mm | 4 mm | 4 mm | 6 mm |
| TR/TE | 6390/73 | 4.62/2.31 | 4330/72 | 4.37/2.19 |
| FA | 150° | 56° | 150° | 65° |

**Supplementary Table 1. MRI defecography protocol details.**

* performed during rest, squeezing, straining, and defecation.

Abbreviations: MR = magnetic resonance; TSE = turbo spin echo; TruFi = true fase imaging with steady-state free precession; TR = time of repetition; TE = time of echo; FA = flip angle.
