# Supplementary material for: A possible physiological mechanism of rectocele formation in women
Source: Abdom Radiol (NY). 2023 Feb 6;48(4):1203–14. doi: 10.1007/s00261-023-03807-2 (PMC10115871; doi:10.1007/s00261-023-03807-2)
Supplement: Supplementary file 2 — Supplementary file2 (DOCX 16 kb) [file 261_2023_3807_MOESM2_ESM.docx]

| **Supplementary Table 2. Patient characteristics** |  |
| --- | --- |
| **Demographic variables** | **All the cases (N = 32)** |
| Age (years) | 48.78 + 12.70* |
| BMI (kg/m^2^) | 25.81 + 5.14* |
| Time interval between MRI and manometry (months) | 4.5 (0 - 12)^#^ |
| Reason for the test |  |
| Constipation | 29 |
| Faecal incontinence | 2 |
| Suspicion of rectocele | 1 |
| Vaginal delivery |  |
| Yes | 22 |
| No | 9 |
| Missing data | 1 |
| Traumatic births |  |
| Yes | 17 |
| No | 12 |
| Missing data | 3 |
| Caesarean section |  |
| Yes | 5 |
| No | 24 |
| Missing data | 3 |
| **^*^**Mean and standard deviation**; ^#^** Median and range | |
